# Auditory brainstem response asymmetries in older adults: An exploratory study using click and speech stimuli

**DOI:** 10.1371/journal.pone.0251287

**Published:** 2021-05-07

**Authors:** Alejandro Ianiszewski, Adrian Fuente, Jean-Pierre Gagné

**Affiliations:** 1 École d’orthophonie et d’audiologie, Faculté de médecine, Université de Montréal, Montréal, Québec, Canada; 2 Centre de recherche de l’Institut universitaire de gériatrie de Montréal, Montréal, Québec, Canada; Universidad de Chile, CHILE

## Abstract

**Background:**

Some evidence suggests that young adults exhibit a selective laterality of auditory brainstem response (ABR) elicited with speech stimuli. Little is known about such an auditory laterality in older adults.

**Objective:**

The aim of this study was to investigate possible asymmetric auditory brainstem processing between right and left ear presentation in older adults.

**Methods:**

Sixty-two older adults presenting with normal hearing thresholds according to their age and who were native speakers of Quebec French participated in this study. ABR was recorded using click and a 40-ms /da/ syllable. ABR was elicited through monaural right and monaural left stimulation. Latency and amplitude for click-and speech-ABR components were compared between right and left ear presentations. In addition, for the /da/ syllable, a fast Fourier transform analysis of the sustained frequency-following response (FFR) of the vowel was performed along with stimulus-to-response and right-left ear correlation analyses.

**Results:**

No significant differences between right and left ear presentation were found for amplitudes and latencies of the click-ABR components. Significantly shorter latencies for right ear presentation as compared to left ear presentation were observed for onset and offset transient components (V, A and O), sustained components (D and E), and voiced transition components (C) of the speech-ABR. In addition, the spectral amplitude of the fundamental frequency (F0) was significantly larger for the left ear presentation than the right ear presentation.

**Conclusions:**

Results of this study show that older adults with normal hearing exhibit symmetric encoding for click stimuli at the brainstem level between the right and left ear presentation. However, they present with brainstem asymmetries for the encoding of selective stimulus components of the speech-ABR between the right and left ear presentation. The right ear presentation of a /da/ syllable elicited reduced neural timing for both transient and sustained components compared to the left ear. Conversely, a stronger left ear F0 encoding was observed. These findings suggest that at a preattentive, sensory stage of auditory processing, older adults lateralize speech stimuli similarly to young adults.

## Introduction

Subcortical asymmetries between the right and left auditory pathways have been reported in newborns [1–3] and young adults [4–10]. Studies conducted in samples of young adults have found that click stimuli seem to produce similar responses (i.e. amplitude and latency) for the auditory brainstem response (ABR) between right and left ear presentation. However, when using speech-like stimuli (e.g. /da/ syllable) an asymmetric pattern characterized by better encoding of both temporal and frequency components of stimuli for right ear presentation has been systematically reported in young adults [e.g. 7, 8, 11, 12]. Therefore, at a preattentive, sensory stage of auditory processing, acoustic elements of speech are asymmetrically processed between the right and left auditory pathways. Some authors have suggested that asymmetric processing of speech-like stimuli at the subcortical level is expected due to hemispheric specialization for speech processing [e.g. 13, 14]. In other words, it has been suggested that hemispheric lateralization is associated with lateralization of the entire auditory pathway [15, 16] and thus speech-like stimuli are more efficiently processed when presented to the right ear [17, 18].

Several studies have demonstrated that hemispheric lateralization diminishes with age and thus, less differentiation between right and left cortices is likely to be observed in older adults [19–21]. Therefore, if subcortical processing is associated with the pattern of hemispheric lateralization as mentioned above, then it would be expected that older adults exhibit a loss or reduction of the asymmetric subcortical processing of speech-like stimuli. However, little is known about asymmetric auditory processing at the brainstem level in older adults. Vander Werff and Burns [9] and Van Yper et al. [22] found no significant click-ABR latency or amplitude differences between right and left ear presentation in a group of older adults with age-appropriate hearing levels. Similarly, Munro et al. [23] found comparable results (in latency and amplitude) for click-ABR between right and left ear presentation in a group of older adults with age-related symmetrical high-frequency sensorineural hearing loss. Therefore, these results are similar to the results found in young adults. With regards to speech-like stimuli, findings of subcortical laterality of speech encoding have only been reported by Vander Werff and Burns [9]. Specifically, faster temporal encoding for right ear presentation compared to left ear presentation was found for the transient component A of the speech-ABR. Asymmetric processing for the other speech-ABR components were not found. According to Vander Werff and Burns however, relatively few participants contributed to the aforementioned significant result. Terefore, mean differences could have been driven by extreme values (in either ear) rather than by an ear laterality effect. As discussed by the authors, this is particularly plausible given the high similarity of each ear’s waveform when they were visually inspected. Thus, according to Vander Werff and Burns [9] a larger group of older adults is necessary to investigate any effects of brainstem laterality for speech encoding. Consequently, it remains unclear whether or not the pattern observed in young adults (i.e. asymmetric responses for speech-like stimuli at the brainstem level between right and left ear input) is modified in older adults. This piece of information is important because asymmetries in the processing of speech sounds throughout the entire auditory pathway (i.e., from the cochlea to the cortex) appear to be critical for normal speech perception [19]. Rapid temporal information, as conveyed in speech sounds, is preferentially processed in the right ear/left auditory cortex pathway [7, 8, 11, 12, 24, 25], whereas frequency components of sounds have been found to engage the left ear/right auditory cortex more strongly than the opposite pathway [26–29]. Thus, a loss of asymmetric processing between the right and left ear pathways may ultimately affect the ability to effectively process acoustic features of speech [19–21]. Therefore, age-related changes in the asymmetric subcortical processing of speech-like stimuli may, at least partially, explain speech perception difficulties that go beyond sound detection problems, widely observed in older adults [30]. The aim of this study was to investigate auditory brainstem processing asymmetries between right and left ear presentations in healthy older adults. With the aim of controlling for central auditory changes associated with a reduction in audibility, we selected a sample of older adults with hearing thresholds expected according to their age [31].

## Methods

### Sample size calculation

The sample size was calculated based on the data reported by Hornickel et al. [7], who investigated subcortical asymmetry of speech encoding in normal hearing young adults by recording brainstem responses to a 40-ms /da/ syllable, monaurally presented to the right and left ears. The same procedure was applied in this study with a sample of older adults. Hornickel et al. found significant ear asymmetries for temporal and frequency components of a 40-ms /da/ syllable. Ear differences showed small to moderate (0.4–0.5) effect sizes which were estimated using Cohen’s *d*. Thus, to calculate the sample size for the current study, the measure of the effect (*d* = 0.4) reported by Hornickel et al. [7] was chosen. Considering a statistical power of 80%, a *p* < 0.05 as significant (using a two-tailed test), and a 10% of probable loss, the sample size for this study was set for 60 participants.

### Participants

Sixty-two older adults (33 women and 29 men) between the ages of 61 and 90 years (mean and Standard Deviation (DS) = 71.80 and 6.28, respectively) were selected. Participants were recruited from the registry of research participants from the Institut Universitaire de Gériatrie de Montréal (IUGM) as well as via posts and word of mouth. All participants presented with no history of middle-ear infections, neurologic conditions, or major chronic health conditions. They were all right-handed according to the Edinburgh Handedness Inventory [32] and spoke Quebec French as their first language. All participants reported that they spoke a second language (in most cases English). None of the participants spoke a tonal language and none of them reported past or present musical training. They exhibited no visible alterations of the ear canal or tympanic membrane under otoscopic examination, and all had bilateral type-A results [33] for tympanometry. Also, bilateral pure-tone audiometry was conducted with an Interacoustics AC40 clinical audiometer (Interacoustics A/S, Middelfart, Denmark) using ER-3A insert earphones (Etymotic Research, Elk Grove Village, IL, USA). All participants presented with pure-tone thresholds at the tested frequencies (i.e. 250, 500, 1000, 2000, 3000, 4000, 6000 and 8000 Hz) without exceeding the 25^th^ percentile (S1 Table) of the distribution of hearing thresholds obtained from an otologically screened population of similar age and sex (ISO 7029–2000) [31]. Moreover, only participants with symmetric hearing thresholds between both ears were included. This was defined as an interaural pure-tone threshold difference of no more than 10 dB at two or less audiometric frequencies between 250 and 8000 Hz. Finally, participants scored at least 26/30 in the Montreal Cognitive Assessment (MoCA, [34]), suggesting no cognitive impairment. Signed consent forms were obtained from all participants, and all study procedures were reviewed and approved by the ethics committee of the Centre de recherche de l’Institut universitaire de gériatrie de Montréal. Participants received a monetary compensation for their participation.

### Neurophysiologic stimuli and recording parameters

Click-and speech-ABR for right and left ear presentation was elicited and registered using a two-channel Intelligent Hearing System (IHS, Miami, FL, United States) SmartEP module (version 3.95). Electrodes placed at Fz (positive), A1 and A2 (negative), and the forehead (ground) in accordance with the International 10 to 20 system EEG were used for all recordings. Contact impedance was maintained below 5 kΩ, and inter-electrode impedance was maintained below 3 kΩ. Click-ABR was obtained in each ear before recording brainstem responses to the /da/ speech syllable. Stimuli were monaurally presented through unshielded insert earphones (ER-3, Etymotic Research, Elk Grove Village, IL, USA). For the click-ABR, clicks were presented at 80 dB nHL in alternating polarity at a rate of 21.1/sec. Online analysis consisted of artifact rejection at 30 μV and digital filtering from 100 to 3000 Hz. Two blocks of 1,024 artifact-free samples were acquired for each ear in a recording window set from 0 to 12 ms relative to stimulus onset. The two blocks were then combined to obtain a grand average of 2,048 sweeps for each ear.

Speech-ABR was elicited by a 40-ms synthesized /da/ syllable provided by the IHS SmartEP module. The syllable contained an initial noise burst and voiced formant transition with a fundamental frequency (F0) that linearly increased from 103 to 125 Hz with a voicing that began at 5 ms with an onset release burst during the first 10 ms. The first formant frequency (F1) linearly increased from 220 to 720 Hz, whereas the second formant frequency (F2) decreased from 1700 to 1240 Hz over the duration of the stimulus. The third formant frequency (F3) decreased slightly from 2580 to 2500 Hz, whereas the fourth (F4) and fifth (F5) formant frequencies remained constant at 3600 and 4500 Hz, respectively [35]. Although the stimulus does not contain a steady-state portion, it is psychophysically perceived as a consonant-vowel speech syllable [36]. (For a detailed description of the synthesized speech stimulus /da/, refer to Johnson et al. [36], and Kraus & Nicol [37]).

The 40-ms /da/ stimulus was monaurally presented to right and left ears at 80 dB SPL in alternating polarity to minimize stimulus artifact at a rate of 10.9/s. A time window of 71.81 ms (including a 20 ms prestimulus time) and online filter setting of 50–3000 Hz was used for recording. Brainstem responses were then offline bandpass filtered from 70 to 2000 Hz. Trials with artifact exceeding 30 μV were excluded from the average. A grand average of 5,000 (two subaverages of 2,500 sweeps) artifact-free responses were obtained for each ear. This number of artifact-free responses was chosen because it falls between the epochs’ range (1600 to 6400) required to record speech-ABRs with clearly identifiable peaks to the 40-ms /da/ syllable [38].

For all ABR testing, participants were seated in a comfortable reclining chair in a quiet room with lights dimmed. The order of ear presentation for click and speech-ABR was counterbalanced across participants. Both ears were plugged with the insert earphone during the entire recording session, regardless of which ear was stimulated. Participants were asked to relax with their eyes closed.

### Discrete peak measure analysis

For each participant, click-and speech-ABR peaks were manually marked. Peaks I, III, and V of the click-ABR were visually identified for each ear using the average waveform obtained from the two brainstem recordings. Latencies and peak-to-trough amplitudes were obtained for all three main peaks. For the speech-ABR, measurements of both timing and magnitude were utilized to assess the discrete peaks. Speech-ABR peaks were expected to appear 7–8 ms after the corresponding stimulus landmark, which is consistent with the neural transmission time from the ear to the midbrain [35]. Krizman et al. [39] peak picking criteria were used to identify the characteristic seven peaks of the response to the 40-ms /da/. Latencies (after stimulus onset) for brainstem transient and sustained peaks were identified using previously described latency values [40]. Speech-ABR peaks included the onset (V and A); the onset of voicing (C), which is supposed to encode the transition from the aperiodic stop burst to the periodic (voiced) formant transition [36]; the frequency-following response (FFR) [D, E, and F], which corresponds to the voiced portion of the syllable, and offset (O) peaks [37]. Interpeak interval differences for the sustained peaks D-E and E-F, which reflect the period of the stimulus fundamental frequency, were also calculated. Latencies and amplitudes of individual peaks for the speech stimuli were further analyzed using an open-source, MATLAB-based toolbox developed and distributed by Erika Skoe, Trent Nicol, and Nina Kraus from the Auditory Neuroscience Laboratory, Northwestern University (Brainstem Toolbox 2013 [41]). Using this toolbox, visually picked peak latencies (after stimulus onset) and corresponding amplitudes previously obtained were automatically adjusted (within ±2 sampling points) to obtain the absolute minimum and maximum [7, 9, 35].

### Sustained measures analysis

Spectral encoding across the FFR region of the 40-ms /da/ neural response was further analyzed using fast Fourier transform (FFT). The FFR region for the 40-ms /da/ response was defined as the time window between 11.4 and 40.6 ms after stimulus onset, which includes peaks C, D, E, and F. Average spectral amplitude was calculated for three frequency ranges: the fundamental frequency (F0 amp: 103–120 Hz), the first formant (F1 amp: 455–720 Hz), and a higher-frequency region corresponding to the seventh-to-eleventh harmonics of stimulus F0 (HF amp: 721–1154 Hz) [7, 9, 11]. The root mean square (RMS) amplitude for the entire period was also calculated. A cross-correlation technique was used to calculate the stimulus-to-response (SR) correlation for each neural response from right and left ears. In addition, a right-left (RL) ear correlation analysis was also carried out. These techniques quantify to what extent two neural signals are related using standard Pearson’s correlation coefficient (r). One signal is displaced in time relative to the other in order to find the temporal delay (time lag) that one signal must undergo to be maximally correlated with the other [17]. The SR correlation analysis was performed for the FFR region of the stimulus (40-ms /da/: 13–34 ms). The RL correlation analysis was performed over the entire neural response (onset peak and FFR). Sustained measure analysis was also conducted using the above-mentioned Brainstem Toolbox 2013 [41].

### Statistical analysis

Data were analyzed with statistical software R (R Core Team 2020, [42, 43]). The data set included a small proportion of missing values (0.9%), which corresponded to peaks that were deemed not replicable or not reliably above the noise floor. Given the within-subject nature of the independent variable (ear), statistical analyses were conducted considering all complete cases per variable, as opposed to complete cases throughout the whole data set. This granted more power to observe differences, since conducting analyses with only complete cases throughout the data set meant working with a substantially reduced sample [44], but involved working with different sample sizes across dependent variables. Using Shapiro-Wilk tests, the first step was to determine whether continuous quantitative variables of interest were normally distributed. Then, normally distributed variables were compared (between ears) conducting paired t-tests, while non-normally distributed variables were compared using Wilcoxon signed-rank tests. Cohen’s *d* and Rosenthal’s formula [45] r = Z/√*N* were reported as effect size measures for these tests to better gauge effects. If statistically significant differences were identified between ears, a second analysis was conducted to determine whether those significant differences were influenced by the participants’ age and/or the magnitude of hearing sensitivity. The latter was defined as the binaural average of pure-tone thresholds at 250, 500, 1000, 2000, 3000, 4000, 6000 and 8000 Hz (binaural PTA). The binaural PTA is expressed by the following formula: [(right ear threshold at 250 + 500 + 1000 + 2000 + 3000 + 4000 + 6000 + 8000 Hz) + (left ear threshold at 250 + 500 + 1000 + 2000 + 3000 + 4000 + 6000 + 8000 Hz)]/16]. Then, multiple mixed-effects models were implemented (Restricted Maximum Likelihood method), with each statistically significant speech-ABR component as dependent variable, participants as a random effect, and ear, age, and binaural PTA as predictors. Following the decision made by the authors of the R package used for these analyses [43] significance for implemented models’ coefficients was based on t-values. Thus, they were significant whenever they exceeded a standard +- 1.96 critical value. Assumptions for all models were inspected, with no major violations detected [46].

## Results

### Pure-tone audiometry results

**Fig 1:**
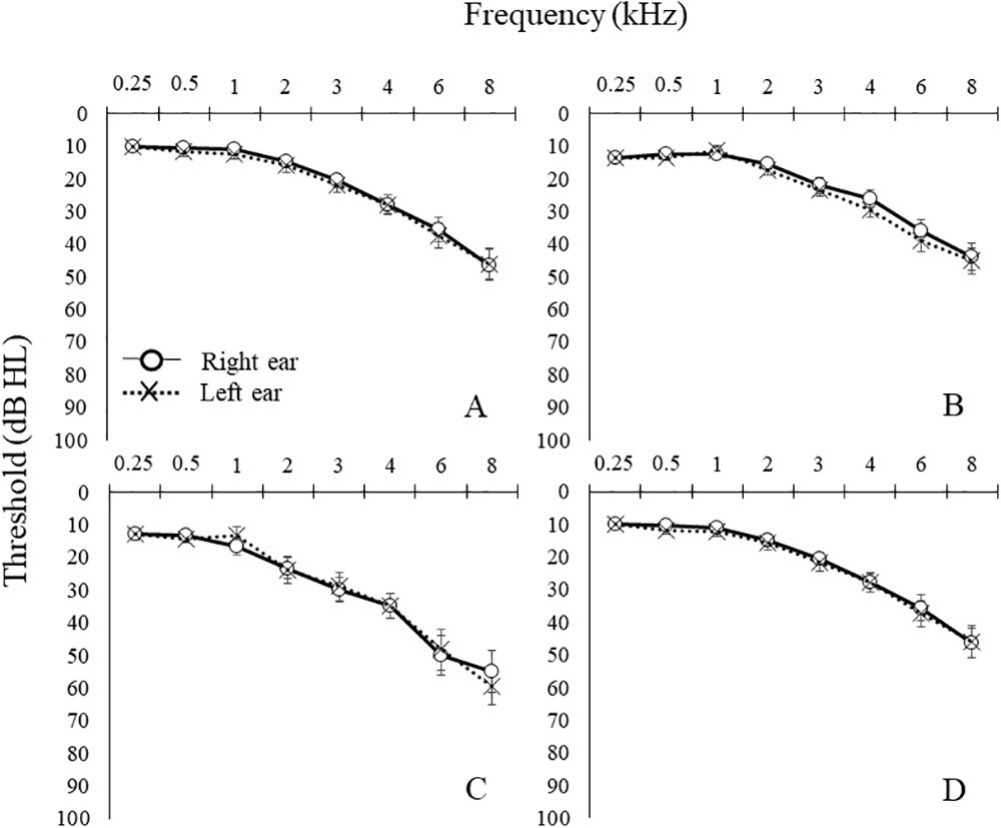
Mean pure-tone thresholds in dB HL for the group of older adults. (A) 60–69 years (n = 23), (B) 70–79 years (n = 31), (C) 80–89 years (n = 8) and (D) grand average (n = 62). Error bars are ±1 SE from the mean. Conventional symbols are used to show data from the right and left ears.

Fig 1 displays the mean and standard errors for pure-tone thresholds for the right and left ears for different age ranges (60–69, 70–79, and 80–89) in the group of participants. No significant differences (p > 0.05) between right and left ear pure-tone thresholds were found across the frequency range (250–8000 Hz). In addition, there was no significant difference for the pure-tone average (p = 0.088) between ears.

### Click-ABR

**Fig 2:**
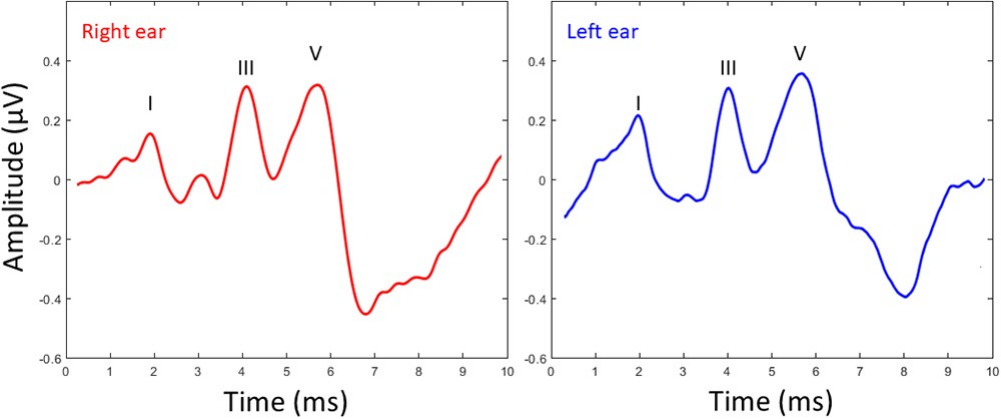
Grand average waveform for the click-ABR obtained from 62 older adults to right (red line) and left (blue line) ear presentation. The stimulus evoked three prominent peaks labeled as I, III and V.

Mean latency and amplitude values for waves I, III, and V of the click-ABR for both ears are shown in Table 1. No significant differences (p > 0.05) between the right and left ears were observed for the latency or amplitude of peaks I, III, and V. More than half of the participants (57.1%) showed a shorter click-ABR wave V latency for right ear stimulation than left ear stimulation, whereas 33.9% of participants showed the opposite pattern. Finally, 8.9% of participants did not present with interaural differences for click-ABR wave V latency. Fig 2 depicts the grand average waveform for the click-ABR for right and left ear presentation.

**Table 1:** Mean and standard deviation (SD) of the latency (after stimulus onset) and amplitude values of the click-ABR components for the right and left ears for the group of participants.

*Measure*	*Ear*				*Test*			
	*Right (SD)*	*%*	*Left (SD)*	*%*	*t*	*Z*	*p-value*	*Effect size*
**Latency (ms)**								
I (n = 44)	1.80 (0.13)	85.5	1.81 (0.15)	75.8	-0.914		0.366	0.137
III (n = 41)	4.00 (0.13)	72.6	3.99 (0.18)	66.1	0.154		0.878	0.024
V (n = 56)	5.87 (0.26)	93.5	5.91 (0.28)	90.3	-1.331		0.189	0.177
**Amplitude (μ*V*)**								
I (n = 44)	0.08 (0.06)		0.10 (0.09)			-0.555	0.579	0.059
III (n = 41)	0.17 (0.08)		0.17 (0.10)		-0.914		0.913	0.017
V (n = 56)	0.34 (0.16)		0.29 (0.19)			-1.731	0.083	0.163

% = percentage of detectability; t = paired t-test; Z = Wilcoxon signed-rank test.

### Speech-ABR

#### Detectability

**Fig 3:**
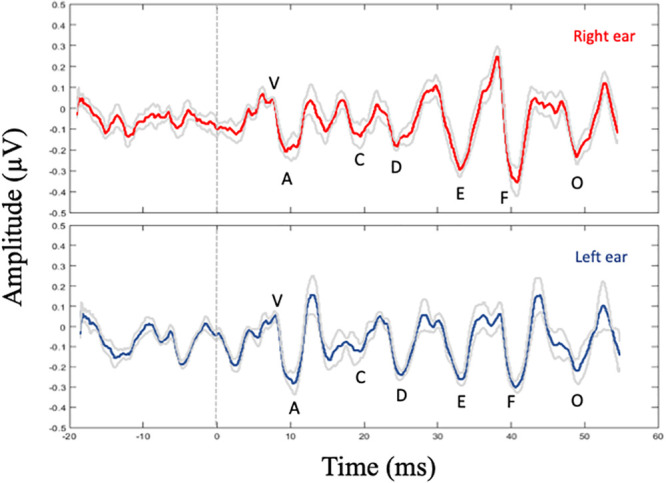
Grand average waveform for the speech-ABR obtained from 62 older adults to right (red line) and left (blue line) ear presentation of 40-ms speech syllable /da/. The stimulus evoked seven prominent peaks, labeled as V, A, C, D, E, F and O. The light-gray lines represent ±1 SE of the mean for the left and the right ears for the entire group of older adults.

Overall, detectability was robust for all peaks, except for peak C, which was detected for 83.9% of the participants in the right ear (52/62 ears) and in 72.6% of the participants in the left ear (45/62 ears). For onset and offset peaks (V-A and O, respectively) detectability varied between 93.5 and 100% in the right ear and between 90.3 and 100% in the left ear. Finally, for the FFR components D, E, and F, detectability varied between 98.4 and 100% in the right ear and between 93.5 and 100% in the left ear. Grand average speech-ABR waveforms for right and left ear presentations are shown in Fig 3.

#### Right and left ear differences for peak latencies and amplitudes

Latency and amplitude values for all the main speech-ABR peaks using the 40-ms /da/ syllable for the right and left ears are displayed in Table 2. Note that ear comparisons were carried out using only the data from those participants whose speech-ABR latencies and amplitudes were identified in both the right and the left ears. Significant ear differences in latency were found for the onset peak V (*t*_*52*_ = -2.674; *p* = 0.010; *d* = 0.367), peak A (*t*_*53*_ = -3.258; *p* = 0.002; *d* = 0.443), and offset peak O (*t*_*61*_ = -4.326; *p* < 0.001; *d* = 0.549). Latency for all transient peaks (V, A, and O) in the right ear were significantly shorter than in the left ear. With regard to peak C, the right ear showed a significantly shorter latency than the left ear (*t*_*39*_ = -2.649; *p* = 0.012; *d* = 0.418). Regarding the FFR components, right ear latencies were also significantly shorter than those for the left ear for components D (*t*_*56*_ = -3.040; *p* = 0.004; *d* = 0.402) and E (*t*_*57*_ = -3.050; *p* = 0.003; *d* = 0.400). No significant latency differences (p > 0.05) were found between ears for peak F. Similarly, no significant interpeak interval differences (p > 0.05) for the sustained peaks D-E and E-F were found. The percentage of participants showing shorter speech-ABR peak latency response for right ear presentation is shown in S2 Table. Finally, no significant differences (p > 0.05) for peak amplitudes between the right and left ears were found (Table 2).

**Table 2:** Mean and standard deviation of latency (after stimulus onset) and amplitude values of the speech-ABR components for the right and left ears for the group of participants.

*Measure*	*Ear*				*Test*			
	*Right (SD)*	*%*	*Left (SD)*	*%*	*t*	*Z*	*p-value*	*Effect size*
**Latency (ms)**								
V (n = 53)	7.00 (0.47)	93.5	7.21 (0.46)	90.3	-2.674		0.010*	0.367
A (n = 54)	8.02 (0.55)	95.2	8.32 (0.67)	90.3	-3.258		0.002**	0.443
C (n = 40)	18.46 (0.72)	83.9	18.87 (0.87)	72.6	-2.649		0.012*	0.418
D (n = 57)	23.80 (0.88)	98.4	24.23 (0.78)	93.5	-3.040		0.004**	0.402
E (n = 58)	31.66 (0.95)	95.2	32.15 (1.11)	96.8	-3.050		0.003**	0.400
F (n = 62)	40.72 (0.92)	100	40.95 (0.89)	100	-1.583		0.119	0.201
O (n = 62)	48.96 (1.22)	100	49.65 (1.13)	100	-4.326		0.000***	0.549
D-E (n = 54)	7.89 (1.02)		7.77 (1.23)		0.538		0.593	0.075
E-F (n = 56)	8.99 (0.95)		8.82 (1.08)		0.996		0.324	0.133
**Amplitude (μ*V)***								
V (n = 53)	0.16 (0.14)		0.21 (0.21)		-1.450		0.153	0.197
A (n = 54)	-0.19 (0.15)		-0.23 (0.20)		1.265		0.211	0.168
C (n = 40)	-0.26 (0.22)		-0.24 (0.34)			-0.430	0.667	0.046
D (n = 57)	-0.24 (0.22)		-0.30 (0.26)			-1.526	0.127	0.142
E (n = 58)	-0.19 (0.27)		-0.21 (0.33)			-0.285	0.776	0.027
F (n = 62)	-0.32 (0.31)		-0.36 (0.35)			-0.998	0.318	0.090
O (n = 62)	-0.24 (0.23)		-0.32 (0.33)			-1.588	0.112	0.142

% = percentage of detectability; t = Paired t-test; Z = Wilcoxon signed-rank test.

*p < 0.05;

**p < 0.01;

***p < 0.001.

#### Stimulus-to-response (SR) and right-and left-ear (RL) response correlations

SR and RL correlation values are reported in Table 3. The maximum SR correlation did not differ significantly (p > 0.05) between both ears. The associated lag between the stimulus and the response, which is based on the time-shifting necessary to obtain the highest correlation was delayed by 0.02 ms in the left ear as compared to the right ear. However, such a delay was not statistically significant (p > 0.05). Regarding the RL response correlation, the left ear response lagged by 0.01 ms as compared to the right ear response relative to the obtained maximum correlation coefficient between both ear responses.

**Table 3:** Mean and standard deviation (SD) values of (A) spectral magnitude measures, (B) stimulus-to-response correlation for right and left ears and (C) right-left correlation for all participants (n = 62).

*Measure*	*Right ear (SD)*	*Left ear (SD)*	*Z*	*p-value*	*Effect size*
**A**. **Spectral magnitudes (μ*V*)**					
RMS	0.48 (0.28)	0.55 (0.31)	-1.932	0.053	0.174
F0	0.34 (0.28)	0.45 (0.35)	-2.433	0.015*	0.218
F1	0.03 (0.01)	0.03 (0.01)	-0.438	0.661	0.039
HF	0.01 (0.003)	0.01 (0.003)	-0.991	0.322	0.088
**B**. **Stimulus-to-response (SR) correlation**					
SR corr (r)	0.14 (0.06)	0.12 (0.07)	-1.515	0.130	0.136
SR lag (ms)	7.97 (1.08)	7.99 (1.12)	-0.099	0.921	0.008
**C**. **Right-left (RL) correlation**					
Straight correlation (Zero lag)	0.071 (0.25)				
Maximum correlation (r)	0.379 (0.19)				
RL lag (ms)	0.011 (1.18)				

Z = Wilcoxon signed-rank test; RMS = Root mean square; F0 = Fundamental frequency; F1 = First formant; HF = Higher frequency formants.

*p < 0.05.

#### Spectral encoding measures

The sustained components of the FFR were also analyzed to investigate possible ear differences in neural phase-locking to the frequency components (Table 3). The overall RMS magnitude for the 40-ms /da/ stimulus was not statistically different (p > 0.05) between both ears. The left ear showed a significantly larger spectral amplitude for the frequency region around the F0 than the right ear (*Z* = -2.433; *p* = 0.015; *r* = 0.218). A 61.3% of participants showed a larger F0 amplitude response for left ear presentation, while 38.7% of participants exhibited either symmetric F0 encoding or a larger amplitude response for right ear presentation. The spectral magnitudes from the other harmonic components did not show significant differences (p > 0.05) between both ears.

#### Effect of age and audibility on ear asymmetries for the speech-ABR

Further analyses were conducted with the seven dependent variables (i.e., speech-ABR peaks V, A, C, D, E, O, and F0 amplitude) which showed statistically significant differences between ears. In order to control for the influence of age and binaural PTA, several mixed-effects models (REML method) were implemented, always specifying participants as a random effect variable. First, the impact of age and binaural PTA was inspected by constructing single-predictor models, with each variable individually predicting all statistically significant speech-ABR components. None of the models showed a significant impact of either variable on any of the observed dependent variables. Second, model comparisons were implemented with Likelihod Ratio tests, comparing a baseline model including age and binaural PTA against a model which added ear as predictor. For all dependent variables, the inclusion of ear significantly improved prediction (chi-squared value always p< 0.05). Table 4 provides a summary of the full models’ results.

**Table 4:** Mixed effects results for all inspected speech-ABR components.

	Estimate	SE	t
**V (n = 53)**			
Age	-0.01	0.01	-1.05
Binaural PTA	-0.01	0.01	-0.94
Ear	-0.20	0.08	-2.62*
**A (n = 54)**			
Age	-0.00	0.01	-0.09
Binaural PTA	-0.01	0.01	-1.73
Ear	-0.30	0.09	-3.26*
**C (n = 40)**			
Age	0.02	0.01	1.65
Binaural PTA	0.00	0.01	0.21
Ear	-0.43	0.14	-3.15*
**D (n = 57)**			
Age	-0.01	0.01	-0.73
Binaural PTA	-0.00	0.01	-0.89
Ear	-0.36	0.14	-2.50*
**E (n = 58)**			
Age	-0.00	0.01	-0.14
Binaural PTA	0.01	0.01	0.75
Ear	-0.48	0.16	-3.06*
**O (n = 62)**			
Age	-0.00	0.02	-0.21
Binaural PTA	-0.00	0.02	-0.21
Ear	-0.69	0.16	-4.33*
**F0 (n = 62)**			
Age	0.00	0.01	-0.54
Binaural PTA	0.00	0.01	0.57
Ear	-0.11	0.05	-2.08*

Coefficients for predictors listed in Estimate column; coefficients are significant (*) whenever their t value is above or below +- 1.96; SE = Standard Error.

## Discussion

What motivated the current study was the question of whether older adults exhibit auditory brainstem processing asymmetries between right and left ear presentation similar to young adults. In a cross-sectional sample of older adults with age-appropriate hearing, our results showed a right ear laterality for selective stimulus components of the speech-ABR. Specifically, significantly shorter latencies were found for the onset (V, A) and offset (O) peaks as well as for some sustained components (C, D, and E) of the speech-ABR elicited from right ear presentation compared to left ear presentation. Moreover, the spectral amplitude of the F0 for the sustained component of the speech-ABR was significantly larger for the left ear than for the right ear presentation. This finding has not been previously reported in either young or older adults. According to our results, none of the above-mentioned findings were modulated either by age or by the magnitude of hearing sensitivity. Shorter latencies and lower spectral amplitude for the frequency region around the F0 in the right ear are consistent with the evidence suggesting that the right ear/left hemisphere pathway is more efficient to process fast temporal modulations, rather than frequency components of sounds [25, 28, 29]. In fact, we hypothesize that the F0 was preferentially encoded by the left ear pathway in the majority of the participants as this pathway has direct access to the right hemisphere which has been associated with the processing of suprasegmental features of speech [26–29, 47, 48]. This hypothesis is further discussed below. The click-ABR showed no significant differences between right and left ear presentation. Overall, latencies for the speech-ABR components found in this sample of older adults were slightly longer than the values reported in young adults [e.g., 7, 8, 11]. However, they were similar to the latency values reported by Vander Werff and Burns [9] and Skoe et al. [40] in older adults. Table 5 provides a comparison of the mean latency values for the speech-ABR components, including studies conducted in both young and older adults.

**Table 5:** Comparative mean and standard deviation of latency values for speech-ABR components using a 40-ms speech syllable /da/ in young and older adults.

Measure	Hornickel et al. (2009) * (YA)		Sinha & Basavaraj (2010) * (YA)		Adahi et al. (2014) * (YA)		Vander Werff & Burns (2011) * (OA)	Skoe et al. (2015) (OA)	Ianiszewski et al. (current study)* (OA)	
	RE (SD)	LE (SD)	RE (SD)	LE (SD)	RE (SD)	LE (SD)	CE (SD)	RE (SD)	RE (SD)	LE (SD)
**Latency (ms)**										
V	6.64 (0.27)	6.58 (0.25)	6.5 (0.26)	6.54 (0.23)	6.72 (0.39)	6.77 (0.41)	6.70 (0.32)	6.92 (0.38)	7.00 (0.47) *	7.21 (0.46) *
A	7.65 (0.38)	7.61 (0.33)	7.36 (0.37)	7.39 (0.35)	7.73 (0.52) *	7.87 (0.52) *	7.75 (0.37) *	7.89 (0.46)	8.02 (0.55)	8.32 (0.67)
C	NR	NR	NR	NR	18.56 (0.69)	18.72 (0.78)	NR	NR	18.46 (0.72) *	18.87 (0.81) *
D	22.52 (0.58) *	22.68 (0.51) *	22.07 (0.69) *	22.68 (0.58) *	22.87 (0.72)	22.91 (0.72)	23.00 (0.87)	23.05 (0.61)	23.80 (0.88) *	24.23 (0.78) *
E	30.96 (0.38)	31.28 (0.58)	30.58 (0.54) *	30.94 (0.55) *	31.50 (1.08) *	31.78 (1.06) *	31.39 (0.91)	31.37 (0.56)	31.66 (0.95) *	32.15 (1.11) *
F	39.33 (0.43) *	39.65 (0.43) *	39.02 (0.53) *	39.45 (0.45) *	40.26 (1.44)	40.39 (1.32)	39.70 (0.56)	39.68 (0.46)	40.72 (0.92)	40.95 (0.89)
O	48.14 (0.39)	48.37 (0.58)	47.43 (0.96) *	47.90 (0.57) *	48.63 (1.03)	48.75 (1.08)	48.70 (0.41)	48.84 (0.56)	48.96 (1.22) *	49.65 (1.13) *

YA = Young adults; OA = Older adults; RE = Right ear; LE = Left ear; CE = Combined ears, NR = Not reported.

* denotes studies and specific peaks where evidence of significant brainstem asymmetries between right and left ear presentation for speech-ABR have been found.

### Asymmetries for the click-ABR in older adults

The results from the click-ABR analysis showed symmetrical responses between the right and left ear presentation. These results are consistent with previous research in older adults [e.g., 9, 10, 22, 23, 49]. Moreover, the evidence of symmetric auditory brainstem processing for click stimuli revealed in this study is also in accordance with findings reported in young adults [4–10]. Therefore, the results from this study along with previous research support the hypothesis of symmetric auditory brainstem processing for click stimuli.

### Asymmetries for the speech-ABR in older adults

The results for the speech-ABR, as opposed to click-ABR, suggest a rather asymmetric auditory brainstem processing for speech acoustic components in older adults. Therefore, it may be suggested that click and speech stimuli elicited different patterns of auditory brainstem activity in this sample of older adults. It is possible that asymmetric processing between both auditory pathways in response to speech sounds results from active exposure to the complex acoustic properties conveyed (e.g., phonetic information) in speech. Hearing speech sounds, as opposed to click sounds, lead to the extraction of significant information about encoding of the time-varying aspect of sounds, which may potentially shape the auditory system to react differently when encoding complex (i.e. speech) versus non-complex (i.e. click) sounds [50]. Moreover, the degree of asymmetry at the brainstem level seems to change accordingly to the complexity of the acoustic signal that needs to be processed [51]. Therefore, pervasive exposure to the complex acoustic sounds and everyday use of speech instead of clicks may reinforce brainstem projections to process more accurately and rapidly the acoustic features of the speech stimulus [7, 52]. Supporting evidence for this hypothesis comes from studies conducted in young adults. A number of studies have found symmetric processing for click-ABR and asymmetric processing for speech-ABR between right and left ear presentation in young adults [7, 8]. There is only one study previously published reporting symmetric processing for click-ABR and asymmetric processing for speech-ABR among older adults [9]. Similar to the findings of this study, Vander Werff and Burns [9] found symmetric processing for click-ABR and an asymmetric processing only for the transient component A of the speech-ABR between right and left ear presentation. Asymmetric processing for the other speech-ABR components were not found. Note that older adults in Vander Werff and Burns’ study showed better hearing thresholds than those obtained by the participants in our study. Although the magnitude of hearing sensitivity did not explain our results, it is possible that hearing thresholds could have accounted for the difference in results between Vander Werff and Burns’ study [9] and our current study. This hypothesis should be further explored. Therefore, taking the above-mentioned studies into account we conclude that older adults, similar to young adults, exhibit a symmetric auditory brainstem processing for click stimuli but an asymmetric auditory brainstem processing for speech-like stimuli.

Note that the results of the speech-ABR found in this study revealed a different pattern of brainstem lateralization for the temporal and frequency acoustic elements of the speech stimulus. Participants, as a group, showed faster temporal encoding for transient and sustained components for right ear presentation and better F0 encoding for left ear presentation. Faster neural timing favoring right ear presentation is consistent with previous findings in young adults showing shorter right ear latencies for speech-ABR transient and sustained components [7, 8, 11]. Thus, older adults investigated in this study showed a rightward laterality of brainstem auditory processing for temporal components of speech, similar to young adults. These results may be attributed to the right ear/left hemisphere pathway specialization for processing complex, rapidly changing acoustic stimuli with a high degree of temporal precision [29, 53, 54].

However, in the frequency domain, the F0 spectral amplitude was significantly larger for the left ear neural response compared to the right ear. This finding is consistent with previous studies showing that frequency components of speech sounds that convey nonlinguistic (suprasegmental) information, such as F0, are more efficiently processed by the left ear/right hemisphere pathway than the opposite pathway [17, 26–29, 42, 43]. Note that in Indo-European languages, such as English or French, F0 does not convey linguistic and/or semantic information, as opposed to tonal languages, in which variations in F0 produce changes in the meaning of the word and are known as lexical tones [55, 56]. Therefore, a larger F0 spectral amplitude for left ear presentation was expected based on the aforementioned theoretical model, as none of the participants spoke a tonal language.

Nevertheless, previous studies of young adults have achieved different and rather contradictory results. On one hand, Hornickel et al. [7] and Ahadi et al. [11] found symmetric encoding of F0 between right and left ear presentation for the same speech stimulus used in the present study in English speakers and monolingual Persian speakers, respectively. Hornickel et al. [7] did not report whether the participants spoke a second language. On the other hand, Sinha and Basavaraj [8], using the same stimulus as the present study’s, found that the F0 spectral amplitude in young adults was significantly larger for the right ear presentation than the left ear presentation. The authors did not report the participants’ native language nor whether they were monolinguals or bilinguals. However, as the study was conducted in India, it may be assumed that the majority of the participants spoke more than one language [57–59]. As mentioned above, our results showed that the older adults in this study, as a group, exhibited a larger F0 for the left ear input than the right ear input. However, individual data showed that the F0 spectral amplitude was larger for left ear presentation in 61.3% of participants, while 37.1% of them exhibited a larger F0 spectral amplitude for right ear presentation, and 1.6% showed no lateralization of F0 encoding (i.e., the same F0 spectral amplitude in both ears). Thus, some of our results are similar to those reported in young adults. Note that as the above-mentioned studies in young adults did not report individual data, it cannot be concluded whether some participants in those studies exhibited an F0 lateralization pattern different than the group mean. Previous researchers [7, 8, 11] have suggested that the 40-ms /da/ syllable used in the studies, including the current one, may be too transient to allow a valid pitch encoding, and thus, left ear preference should not be attained. This hypothesis explains the group results for studies carried out in young adults and for around 38% of the participants in the present study.

We cannot fully explain why around 61% of the participants in this study exhibited a preferential F0 spectral amplitude for left ear presentation, as compared to previous studies conducted in young adults. We propose three hypotheses for this finding. First, participants who exhibited a larger F0 spectral amplitude for left ear presentation may have indeed been able to perceive the brief periodic portion of the stimulus as a tone rather than a transient, enabling them to process F0 as a suprasegmental component of the stimulus. However, to accept this hypothesis, the majority of the participants in this research should have presented with a distinct characteristic that is not found in the samples of young adults investigated in previous research. Certainly, the age of the participants is an important difference between this sample and the previous samples investigated. Nevertheless, around 37% of the participants in this sample, even if their age was similar to the remaining 61%, exhibited an F0 spectral amplitude similar to the findings reported in young adults (e.g., larger for right ear input). Thus, we believe that aging itself cannot account for this particular result. A possible explanation for this finding can be bilingualism. Note that all participants in this study lived in Montreal, which is a bilingual city where people are exposed to English and French at different levels—while some people may solely utilize one language in everyday life with little contact with the other language, others may be exposed to and use both languages on a regular basis. All participants in this study reported that they spoke two languages (in most of the cases, French and English). It has been previously suggested that bilingualism is associated with enhanced neural encoding of speech sounds at the brainstem level [60–62], and recent data using the FFR have shown that bilingual listeners exhibit better encoding of acoustic features of speech than their monolingual peers [63, 64]. Therefore, we hypothesize that enhanced neural encoding for speech sounds induced by bilingualism may have been associated with the capacity to extract F0 as a suprasegmental aspect of the /da/ syllable and thus triggered left ear/right hemisphere preferential processing, as discussed above. However, with the current data, we cannot test this hypothesis, as (a) we did not determine the degree of bilingualism in each participant, and (b) previous research in young adults did not report whether participants spoke more than one language. In addition, no previous studies have investigated differences for the encoding of F0 between right and left ear presentation using the FFR comparing monolingual and bilingual speakers. Thus, we cannot determine whether those participants who exhibited larger F0 spectral amplitudes for left ear presentation (i.e., 61% of the sample) differed in terms of their bilingual experience from the other participants. Future studies should be conducted to test this hypothesis. Second, it may be possible that biological variability accounts for the differences observed. For the short speech-like stimulus (/da/), some listeners are simply able to extract F0 as a suprasegmental aspect of the stimulus, and others are not able to do so. This can be explained by the variability we observed in the participants of this study regarding the lateralization of F0. As mentioned above, previous studies have not reported the percentages of listeners with larger right or larger left F0 spectral amplitudes. Therefore, previous results may just represent the group trend without necessarily representing individual results. Thus, further studies in this field need to be carried out with the aim to test this hypothesis. In addition, we suggest that future studies should report the percentage of listeners who exhibit larger F0 amplitudes for the right and left ears. Third, we also consider the possibility of a technical bias due to electrode montage [65]. Electrode placement [e.g., 66–68] can affect the amplitude of the auditory brainstem response, biasing enhanced amplitude towards one ear. If this bias occurred, we do not believe that it completely accounted for the larger F0 amplitude in 61.3% of the participants. Finally, it may be possible that each of these hypotheses is not exclusive, and thus, a combination thereof may have triggered these results.

In summary, this study suggests an asymmetric auditory brainstem processing between right and left ear presentation of speech-like stimuli. In this sample of older adults, a distinct pattern characterized by a larger F0 spectral amplitude of the 40-ms /da/ syllable for left ear presentation was observed, as opposed to previous studies conducted in young adults. Further research is required to better understand this finding, especially the effect of bilingualism on the capacity of the auditory system to extract and process F0 in short speech-like stimulus.

## Limitations of the study

There were a few caveats that should be considered for future research. First, lifelong experience such as music and bilingualism may enhance neural encoding of complex sound features such as neural timing and frequency encoding [62–64, 69, 70]. Although none of the participants reported past or present musical training, they were all bilingual speakers. Therefore, bilingual experience might have enhanced FFR neural representation of speech components in older adults. Future studies should investigate whether music and bilingualism may modulate the pattern of subcortical laterality of speech encoding among older adults. Second, participants were mainly selected from a registry of participants who are actively involved in research. Therefore, given their profile, they may not represent the general population of older adults. Third, given the difficulty to identify some of the click-and speech-ABR peaks in certain participants, some statistical analyses (e.g., ear comparisons for click-ABR peaks I and III and speech-ABR peak C) were carried out with a number of observations lower than those established according to the sample size calculation Therefore, caution is warranted to interprete the aforementioned results. Fourth, although our detectability percentage for the different speech-ABR components was rather high, we observed inter-individual variations in the response. Some of the neural responses showed patterns of complex morphology. This may be attributed to background noise contamination or muscle artifact.

## Conclusions

The current study presents data to support brainstem laterality for the encoding of acoustic components of speech in older adults. In addition, no asymmetric brainstem processing for click stimuli was found in the sample of older adults. Overall, both findings suggest that older adults with age-appropriate hearing exhibit a pattern of brainstem laterality of click and speech encoding similar to young adults. A result that has not previously been reported in either young or older adults is the larger spectral F0 amplitude (for the 40-ms /da/ syllable) for left ear presentation as opposed to right ear presentation, which suggests that the majority of the listeners were capable of perceiving the very short periodic component of the stimulus as a tone. Future studies should be conducted to further explore this finding and the variables, such as bilingualism, that may be associated with it. Finally, it should be noted that our findings may not be entirely representative of the older adult population. The characteristics (e.g., audibility, cognition, and spoken language) of the sample may not accurately represent the general population of older adults and may have affected the results of this study. Therefore, caution is warranted in generalizing these results to the general population of older adults.

## Supporting information

S1 Table Hearing thresholds corresponding to the 25^th^ percentile for sex and age according to the 7029 ISO standards. (DOCX)

S2 Table Percentage and number (n) of participants showing shorter latency response for right ear presentation, left ear presentation, and no interaural latency difference for each speech-ABR peak. (DOCX)

S1 Data (SAV)

## References

[1] Eldredge L, Salamy A. Functional auditory development in preterm and full-term infants. Early Hum Dev. 1996; 45: 215–228. 10.1016/0378-3782(96)01732-x 8855395

[2] Sininger YS, Cone-Wesson B. Lateral asymmetry in the ABR of neonates: Evidence and mechanisms. Hear Res. 2006; 212: 203–211. 10.1016/j.heares.2005.12.003 16439078

[3] Sininger YS, Cone-Wesson B, Abdala C. Gender distinctions and lateral asymmetry in the low-level auditory brainstem response of the human neonate. Hear Res. 1998; 126: 58–66. 10.1016/s0378-5955(98)00152-x 9872134

[4] Hixson C, Mosko M. Normative bilateral brainstem evoked response data for a naval aviation student population: Groups statistics. Nav Aerosp Med Res Lab Pensacola Fla. 1978. 27–28.

[5] Rowe J. Norma variability of the brain-stem auditory evoked response in young and old adult subjects. Electroencephalography and Clin Neurophysiol. 1978; 44: 459–470. 10.1016/0013-4694(78)90030-5 76554

[6] Lauter J, Karzon R. Individual Differences in Auditory Electric Responses III. A Replication, with Observations of Individual vs. Group Characteristics. Scandinavian audiology. 1990; 19 (2):67–72. 10.3109/01050399009070755 2371537

[7] Hornickel J, Skoe E, Kraus N. Subcortical laterality of speech encoding. Audiol Neurootol. 2009; 14(3): 198–207. 10.1159/000188533 19122453 PMC2806639

[8] Sinha K, Basavaraj V. Lateral asymmetry in speech processing at the brainstem: evidence from speech evoked ABR. JAIISH. 2010; 29(1):101–109.

[9] Vander Werff KR, Burns K. Brain stem responses to speech in younger and older adults. Ear Hear. 2011; 32: 168–179. 10.1097/AUD.0b013e3181f534b5 21052004

[10] Peng L, Yu S, Jing Y, Chen R, Liang J. Diffusion tensor imaging of the central auditory system in the elderly. Lin Chung Er Bi Yan Hou Tou Jing Wai Ke Za Zhi. 2016; 30 (8): 637–640. 10.13201/j.issn.1001-1781.2016.08.014 29871096

[11] Ahadi M, Pourbakht A, Jafari A, Jalaie S. Effects of stimulus presentation mode and subcortical laterality in speech evoked auditory brainstem responses, International Journal of Audiology. 2014; 53(4): 243–249. 10.3109/14992027.2013.866281 24506562

[12] Krishnan A, Gandour J, Krishnan S, Bidelman G, Smalt Ch. Functional ear (a)symmetry in brainstem neural activity relevant to encoding of voice pitch: A precursor for hemispheric specialization? Brain & Language. 2011; 119: 226–231. 10.1016/j.bandl.2011.05.001 21658753 PMC3193894

[13] Levine R, McGaffigan P. Right-left asymmetries in the human brain stem: auditory evoked potentials. Electroencephalography and Clin Neurophysiol. 1983; 55: 532–537. 10.1016/0013-4694(83)90163-3 6187546

[14] Levine R, Liederman J, Riley P. The brainstem auditory evoked potential asymmetry is replicable and reliable. Neuropsychologia. 1988; 26(4): 603–614. 10.1016/0028-3932(88)90116-9 3405403

[15] Schönwiesner M, Krumbholz K, Rübsamen R, Fink G, & von Cramon D. Hemispheric asymmetry for auditory processing in the human auditory brain stem, thalamus, and cortex. Cerebral Cortex. 2007; 17(2): 492–499. 10.1093/cercor/bhj165 16565292

[16] Philibert B, Veuillet E, & Collet L. Functional asymmetries of crossed and uncrossed medial olivocochlear efferent pathways in humans. Neuroscience letters. 1998; 253(2): 99–102. 10.1016/s0304-3940(98)00615-6 9774159

[17] Jerger J, & Martin J. Hemispheric asymmetry of the right ear advantage in dichotic listening. Hearing research. 2004; 198(1–2): 125–136. 10.1016/j.heares.2004.07.019 15567609

[18] Kimura D. From ear to brain. Brain and Cognition. 2011; 76(2): 214–217. 10.1016/j.bandc.2010.11.009 21236541

[19] Bellis TJ, Nicol T, Kraus N. Aging affects the hemispheric asymmetry in the neural representation of speech sounds. Journal of Neuroscience. 2000; 20: 791–797. 10.1523/JNEUROSCI.20-02-00791.2000 10632608 PMC6772399

[20] Goossens T, Vercammen C, Wouters J, Van Wieringen A. Aging Affects Neural Synchronization to Speech-Related Acoustic Modulations. Front. Aging Neurosci. 2016; 8:133. 10.3389/fnagi.2016.00133 27378906 PMC4908923

[21] Chen X, Liang Y, Deng Y, Li J, Chen S, Wang C., et al. Age- associated reduction of asymmetry in human central auditory function: a 1H-magnetic resonance spectroscopy study. Neural Plast. 2013; 1–7. 10.1155/2013/735290 24222864 PMC3809597

[22] Van Yper LN, Vermeire K, De Vel EF, Beynon AJ, Dhooge I. Age-Related Changes in Binaural Interaction at Brainstem Level. Ear Hear. 2016; 37(4): 434–442. 10.1097/AUD.0000000000000274 26881979

[23] Munro K, Pisareva N, Parker D, Purdy S. Asymmetry in the auditory brainstem response following experience of monaural amplification. Neuro Report. 2007; 18:1871–1874. 10.1097/WNR.0b013e3282f1b003 18090329

[24] Belin P, Zilbovicius M, Crozier S, Thivard L, Fontaine A, Masure M-C, et al. Lateralization of speech and auditory temporal processing. J Cognit Neurosci. 1998; 10:536–540. 9712682 10.1162/089892998562834

[25] Tervaniemi M, Hugdahl K. Lateralization of auditory-cortex functions. Brain Res Rev. 2003; 43(3): 231–246. 10.1016/j.brainresrev.2003.08.004 14629926

[26] Ballachanda B, Rupert A, Moushegian G. Asymmetric frequency following responses. J Am Acad Audiol. 1994; 5: 133–137. 8180429

[27] Ballachanda B, Moushegian G. Frequency-following response: Effects of interaural time and intensity differences. J Am Acad Audiol. 2000; 11: 1. 10741352

[28] Zatorre R, Evans A, Meyer E, Gjedde A. Lateralization of phonetic and pitch discrimination in speech processing. Science. 1992; 256 (5058):846–9. 10.1126/science.1589767 1589767

[29] Zatorre R, Belin P. Spectral and temporal processing in human auditory cortex. Cereb Cortex. 2001; 11, 946–953. 10.1093/cercor/11.10.946 11549617

[30] Humes L, Dubno J, Gordon-Salant S, Lister J, Cacace A, Cruickshanks K, et al. Central presbycusis: a review and evaluation of the evidence. Journal of the American Academy of Audiology. 2012; 23(8): 635–666. 10.3766/jaaa.23.8.5 22967738 PMC5898229

[31] International Organization for Standardization. Acoustics Statistical Distribution of Hearing Thresholds as a Function of Age. ISO 7029. 2000. Geneva: ISO.

[32] Oldfield R. The assessment and analysis of handedness the Edinburgh inventory. Neuropsychologia. 1971; 9: 97–113. 10.1016/0028-3932(71)90067-4 5146491

[33] Jerger J. Clinical Experience with impedance audiometry. Arch Otolaryng. 1970; 92: 311–324. 10.1001/archotol.1970.04310040005002 5455571

[34] Nasreddine Z, Phillips N, Bédirian V, Charbonneau S, Whitehead V, Collin I, et al. The Montreal Cognitive Assessment, MoCA- A Brief Screening Tool for Mild Cognitive Impairment. Journal of American Geriatrics Society. 2005; 53(4): 695–699. 10.1111/j.1532-5415.2005.53221.x 15817019

[35] Skoe E, Kraus N. Auditory brainstem response to complex sounds: A tutorial. Ear Hear. 2010; 31: 302. 10.1097/AUD.0b013e3181cdb272 20084007 PMC2868335

[36] Johnson KL, Nicol TG, Kraus N. The brainstem response to speech: a biological marker of auditory processing. Ear Hearing. 2005; 26: 424–434. 16230893 10.1097/01.aud.0000179687.71662.6e

[37] Kraus N, Nicol T. Brainstem origins for cortical -what-and-where- pathways in the auditory system. Trends Neuroscience. 2005; 28 (4): 176–181. 10.1016/j.tins.2005.02.003 15808351

[38] BinKhamis G, Léger A, Bell S, Prendergast G, O’Driscoll M, Kluk K. Speech auditory brainstem responses: Effects of background, stimulus duration, consonant–vowel, and number of epochs. Ear and hearing. 2019; 40(3): 659–670 10.1097/AUD.0000000000000648 30124503 PMC6493675

[39] Krizman J, Skoe E, Kraus N. Sex differences in auditory subcortical function. Clin Neurophysiol. 2012; 123: 590–597. 10.1016/j.clinph.2011.07.037 21855407 PMC3226913

[40] Skoe E, Krizman J, Anderson S, Kraus N. Stability and Plasticity of Auditory Brainstem Function Across the Lifespan. Cerebral Cortex. 2015; 25:1415–1426. 10.1093/cercor/bht311 24366906 PMC4428291

[41] Skoe E, Nicol T, Kraus N. The Brainstem Toolbox. Version 2013. www.brainvolts.northwestern.edu.

[42] Team, R. Core. R: A language and environment for statistical computing. Vienna: R Foundation for Statistical Computing. 2020: 201.

[43] Bates D, Maechler M, Bolker B, Walker S. Fitting Linear Mixed-Effects Models Using lme4. Journal of Statistical Software. 2015; 67(1): 1–48.

[44] Pigott TD. A review of methods for missing data. Educational research and evaluation. 2001;7(4):353–83.

[45] Rosenthal R. Parametric measures of effect size. 1994. In Cooper H. & Hedges L. V. (Eds.), The handbook of research synthesis. (pp. 231–244). New York: Russell Sage Foundation.

[46] Schielzeth H, Dingemanse NJ, Nakagawa S, Westneat DF, Allegue H, Teplitsky C, et al. Robustness of linear mixed-effects models to violations of distributional assumptions. Methods in Ecology and Evolution. 2020;11(9):1141–52.

[47] Liégeois-Chauvel C, Giraud K, Badier JM, Marquis P, Chauvel P. Intracerebral evoked potentials in pitch perception reveal a functional asymmetry of the human auditory cortex. Ann NY Acad Sci. 2001; 930:117–132. 10.1111/j.1749-6632.2001.tb05728.x 11458823

[48] Johnsrude I, Penhune I, Zatorre R. Functional specificity in the right human auditory cortex for perceiving pitch direction. Brain, 2000; 123 (1): 155–163. 10.1093/brain/123.1.155 10611129

[49] Johansen HS, Lehn T. The dependence of early acoustically evoked potentials on age. Arch Otorhinolaryngol. 1984; 240:153. 10.1007/BF00453473 6477293

[50] Johnson K, Nicol T, Zecker S, Kraus N. Developmental plasticity in the human auditory brainstem. J Neurosci. 2008; 28: 4000–4007. 10.1523/JNEUROSCI.0012-08.2008 18400899 PMC2806643

[51] King C, Nicol T, McGee T, Kraus N. Thalamic asymmetry is related to acoustic signal complexity. Neurosci Lett. 1999; 267: 89–92. 10.1016/s0304-3940(99)00336-5 10400219

[52] Firszt J, Ulmer J, Gaggl W. Differential Representation of Speech Sounds in the Human Cerebral Hemispheres. Anat Rec A Discov Mol Cell Evol Biol. 2006; 288 (4): 345–357. 10.1002/ar.a.20295 16550560 PMC3780356

[53] Nicholls M, Gora J, Stough C. Hemispheric asymmetries for visual and auditory temporal processing: an evoked potential study. International Journal of Psychophysiology. 2002; 44: 37–55 10.1016/s0167-8760(01)00190-8 11852156

[54] McGettigan C, Scott S. Cortical asymmetries in speech perception: what’s wrong, what’s right, and what’s left? Trends Cogn Sci. 2012; 16 (5): 269–276 10.1016/j.tics.2012.04.006 22521208 PMC4083255

[55] Gandour J. Phonetics of tone. In: Asher R.; Simpson J., editors. The encyclopedia of language & linguistics. New York: Pergamon Press; 1994. p. 3116–3123.

[56] Liu H, Wang EQ, Chen Z, Liu P, Larson CR, Huang D. Effect of tonal native language on voice fundamental frequency responses to pitch feedback perturbations during sustained vocalizations. J Acoust Soc Am. 2010;128(6):3739–3746 10.1121/1.3500675 21218905 PMC3037774

[57] Weinreich U. (1957). Functional aspects of Indian bilingualism. Word, 13(2), 203–233.

[58] Clingingsmith D. (2014). Industrialization and bilingualism in India. Journal of Human Resources, 49(1), 73–109.

[59] Azam M., Chin A., & Prakash N. (2013). The returns to English-language skills in India. Economic Development and Cultural Change, 61(2), 335–367.

[60] Krishnan A, Gandour JT. The role of the auditory brainstem in processing linguistically-relevant pitch patterns. Brain Lang. 2009; 110:135–148. 10.1016/j.bandl.2009.03.005 19366639 PMC2731823

[61] Krishnan A, Gandour J, Bidelman G. Experience-dependent plasticity in pitch encoding: from brainstem to auditory cortex. Neuroreport. 2012; 23 (8): 498. 10.1097/WNR.0b013e328353764d 22495037 PMC3342423

[62] Kraus N, Anderson S. Bilingualism enhances neural speech encoding. The Hearing Journal. 2014; 67 (7): 40.

[63] Skoe E, Burakiewicz E, Figueiredo M, Hardin M. Basic neural processing of sound in adults is influenced by bilingual experience. Neuroscience. 2017; 349: 278–290. 10.1016/j.neuroscience.2017.02.049 28259798

[64] Krizman J, Marian V, Shook A, Skoe E, Kraus N. Subcortical encoding of sound is enhanced in bilinguals and relates to executive function advantages. Proceedings of the National Academy of Sciences. 2012; 109 (20): 7877–7881. 10.1073/pnas.1201575109 22547804 PMC3356657

[65] Hood L. Clinical applications of the auditory brainstem response. Singular, 1998.

[66] Beattie R, Beguwala F, Mills D, Boyd R. Latency and amplitude effects of electrode placement on the early auditory evoked response. Journal of Speech and Hearing Disorder. 1986; 51(1): 63–70 10.1044/jshd.5101.63 3945061

[67] Dzulkarnain A, Wilson W, Bradley A, Petoe M. The effects of electrode montage on the amplitude of wave V in the auditory brainstem response to maximum length sequence stimuli. Audiology and Neurotology. 2008; 13(1): 7–12. 10.1159/000107432 17715464

[68] Dzulkarnain A, Tengku Zam Zam T, Azed Z, Rahman Zuri M, Sulaiman N. Effects of electrode position on tone-burst-evoked auditory brainstem responses (ABR) in humans. Middle-East Journal of Scientific Research. 2014; 21: 1180–1187.

[69] Parbery-Clark A, Anderson S, Hittner E, Kraus N. Musical experience offsets age-related delays in neural timing. Neurobiology of Aging. 2012; 33:1483 10.1016/j.neurobiolaging.2011.12.015 22227006

[70] White-Schwoch T, Carr K, Anderson S, Strait D, Kraus N. Older Adults Benefit from Music Training Early in Life: Biological Evidence for Long-Term Training-Driven Plasticity. The Journal of Neuroscience. 2013; 33(45): 17667–17674. 10.1523/JNEUROSCI.2560-13.2013 24198359 PMC3818545

