# Supplementary material for: Auditory brainstem response asymmetries in older adults: An exploratory study using click and speech stimuli
Source: PLoS One. 2021 May 7;16(5):e0251287. doi: 10.1371/journal.pone.0251287 (PMC8104406; doi:10.1371/journal.pone.0251287)
Supplement: S1 Table — (DOCX) [file pone.0251287.s001.docx]

| **S1 Table.** Hearing thresholds corresponding to the 25^th^ percentile for sex and age according to the 7029 ISO standards. | | | | |
| --- | --- | --- | --- | --- |
|  | 60-69 years | | 70-79 years | |
| Frequency (Hz) | Male (dB HL) | Female (dB HL) | Male (dB HL) | Female (dB HL) |
| 250 | 11 | 11 | 15 | 15 |
| 500 | 12 | 12 | 16 | 16 |
| 1000 | 13 | 13 | 18 | 18 |
| 2000 | 21 | 18 | 30 | 26 |
| 3000 | 32 | 22 | 46 | 31 |
| 4000 | 42 | 26 | 62 | 37 |
| 6000 | 48 | 34 | 70 | 48 |
| 8000 | 58 | 42 | >80 | 60 |
