# Supplementary material for: Auditory brainstem response asymmetries in older adults: An exploratory study using click and speech stimuli
Source: PLoS One. 2021 May 7;16(5):e0251287. doi: 10.1371/journal.pone.0251287 (PMC8104406; doi:10.1371/journal.pone.0251287)
Supplement: S2 Table — (DOCX) [file pone.0251287.s002.docx]

| **S2 Table.** Percentage and number (n) of participants showing shorter latency response for right ear presentation, left ear presentation, and no interaural latency difference for each speech-ABR peak. | | | |
| --- | --- | --- | --- |
|  | *Right ear* | *No interaural latency difference* | *Left ear* |
| **Peaks** | | | |
| V (n = 53) | 50.9 (27) | 13.2 (7) | 35.8 (19) |
| A (n = 54) | 62.9 (34) | 5.6 (3) | 31.5 (17) |
| C (n = 40) | 60.0 (24) | 5.0 (2) | 35.0 (14) |
| D (n = 57) | 68.4 (39) | 3.5 (2) | 28.1 (16) |
| E (n = 58) | 67.2 (39) | 3.4 (2) | 29.3 (17) |
| F (n = 62) | 59.7 (37) | 6.5 (4) | 40.3 (25) |
| O (n = 62) | 75.8 (47) | 1.6 (1) | 22.6 (14) |
